# Correcting for cryptic relatedness by a regression-based genomic control method

**DOI:** 10.1186/1471-2156-10-78

**Published:** 2009-12-02

**Authors:** Ting Yan, Bo Hou, Yaning Yang

**Affiliations:** 1 Department of Statistics and Finance, University of Science and Technology of China, Hefei, Anhui 230026, PR China; 2 Department of Statistics, Fudan University, Shanghai 200433, PR China

## Abstract

**Background:**

Genomic control (GC) method is a useful tool to correct for the cryptic relatedness in population-based association studies. It was originally proposed for correcting for the variance inflation of Cochran-Armitage's additive trend test by using information from unlinked null markers, and was later generalized to be applicable to other tests with the additional requirement that the null markers are matched with the candidate marker in allele frequencies. However, matching allele frequencies limits the number of available null markers and thus limits the applicability of the GC method. On the other hand, errors in genotype/allele frequencies may cause further bias and variance inflation and thereby aggravate the effect of GC correction.

**Results:**

In this paper, we propose a regression-based GC method using null markers that are not necessarily matched in allele frequencies with the candidate marker. Variation of allele frequencies of the null markers is adjusted by a regression method.

**Conclusion:**

The proposed method can be readily applied to the Cochran-Armitage's trend tests other than the additive trend test, the Pearson's chi-square test and other robust efficiency tests. Simulation results show that the proposed method is effective in controlling type I error in the presence of population substructure.

## Background

Population-based genetic association analysis is a powerful method for detecting susceptibility loci for complex diseases. A common issue in such design is that it may be subject to population heterogeneity and, as a result, spurious association may be reported if the population substructure is not properly addressed. Many methods have been proposed to deal with population heterogeneity in genetic association analysis.

When there is population stratification (PS) on allele frequencies, a direct method is to use family-based design [1-5] in which unaffected family members are chosen to match each case so that the association detected is truly due to the linkage between the candidate marker and the disease. But this method is limited by the cost and the difficulty in recruiting family members. Pritchard et al. [6,7] used a Bayesian clustering method to infer the number of subpopulations and to assign the individuals to putative subpopulations. The inferred memberships in each subpopulation are then used to perform tests of association for that subpopulation. A modification of this method was implemented by Satten et al. [8], in which subpopulation memberships were decided by a latent class model. Patterson et al. [9] proposed a principle components analysis method to correct for the population structure and obtained a test statistic based on the eigenvalues of the correlation matrix to detect the population structure. When the population has substructure, the usual chi-square statistics have non-central chi-square distributions under the null. Gorroochurn et al. [10] proposed a δ-centralization method to correct for PS by centralizing the test statistics using information from the null markers.

Another form of population heterogeneity is the cryptic relatedness or correlation across individuals. For this type of data, Devlin and Roeder [11] developed the genomic-control (GC) method to correct for the variance inflation. They proposed to use the additive Cochran-Armitage trend test to detect the gene-phenotype association. Assuming that the correlations or kinship coefficients are the same across all markers, they showed that the scaled test statistic has asymptotically a 1-df chi-square distribution. The scaling factor, known as the variance inflation factor (VIF), can be estimated from information of the unlinked null markers.

The GC method is a simple and effective method in association studies to correct for population heterogeneity caused by cryptic relatedness. However, when the GC method is applied to recessive or dominant trend tests [12] or, to Pearson's chi-square test [13] or other robust tests [14], the null loci are required to match with the candidate loci in allele frequencies, which reduces the number of available null makers.

In this study, we propose a regression-based genomic control (RGC) method that can be applied to association tests other than the additive trend test. This method allows for using arbitrary null markers in the GC correction procedure by adjusting the variability of the allele frequencies of the null markers through linear regression. We use simulation studies to check whether the method appropriately corrects for the problem of spurious association. In addition the robustness of the proposed method to the errors in selecting null markers is assessed. We also simulate the power of our method.

## Methods

### Trend tests

Let *A *be the high-risk candidate allele with the allele frequency *p *and *a *the normal one with the allele frequency *q *= 1 - *p*. To detect the association between the marker *A *and a disease, we assume that there are *n*_0 _cases and *n*_1 _controls with total *n *= *n*_0 _+ *n*_1 _individuals. The genotype data are summarized in Table 1.

**Table 1:** Genotype counts

	Genotype			
		
Group	*aa*	*Aa*	*AA*	Total
Case	*n*_00_	*n*_01_	*n*_02_	*n*_0_
Control	*n*_10_	*n*_11_	*n*_12_	*n*_1_

Total	*m*_0_	*m*_1_	*m*_2_	*n*

Denote the three genotypes by *G*_0 _= *aa*, *G*_1 _= *Aa *and *G*_2 _= *AA*. Let *f*_*i *_= *P*(*case|G*_*i*_) be the penetrance given genotype *G*_*i*_, *i *= 0, 1, 2. The null hypothesis of no association between the candidate marker and a disorder can be expressed as *H*_0_: *f*_0 _= *f*_1 _= *f*_2_. Since *A *is a high risk allele and *a *a normal one, let the score of genotype *aa *be 0, and that of *AA *be 1. For a specific choice of score *x *for genotype *Aa*, let

be the difference in weighted allele frequency between cases and controls, where  = *n*_*ij*_/*n*_*i*_, *i *= 0, 1, *j *= 1, 2. When there is no allelic dependence or Hardy-Weinberg equilibrium holds in the population, Δ_*x *_has, under the null hypothesis, the variance(1)

where *p*_1 _and *p*_2 _are the frequencies of *Aa *and *AA *respectively. It can be estimated by

where  = *m*_*k*_/*n *is the estimate of *p*_*k*_, *k *= 1, 2. The Cochran-Armitage's trend test indexed by *x *is then given by(2)

**Figure 1:**
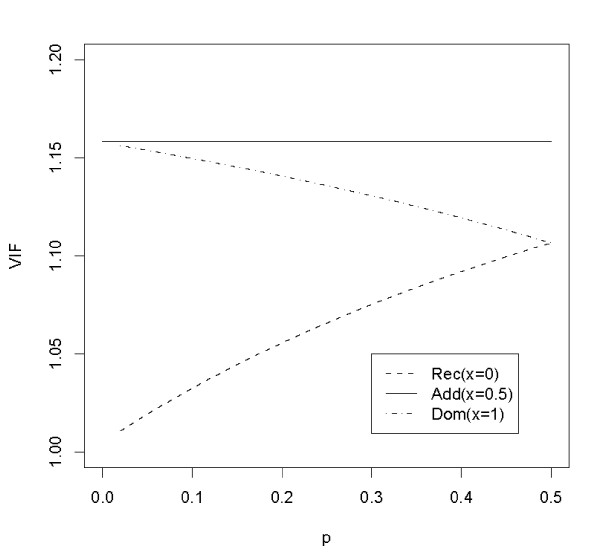
**VIF as function of allele frequency p of candidate marker (F = 0.01)**.

In standard situation,  has a central chi-square distribution with one degree of freedom under the null hypothesis. However, if there is cryptic relatedness,  may be inflated. Denote the inflated variance of Δ_*x *_under the null by  = *var*_*CR*_(Δ_*x*_) and the variance inflation factor by . By this notation, in the presence of CR, *Z*_*x *_~ *N*(0, *λ*_*x*_) under the null hypothesis. Illustrated in Figure 1 are the VIF *λ*_0_, *λ*_0.5 _and *λ*_1 _as a function of the allele frequency *p *of the candidate marker. This figure was drawn from a simulated data of three subpopulations with (20, 30, 50) cases and (50, 30, 20) controls, and the Wright's coefficient *F *being 0.01. It shows that *λ*_0.5 _of the additive model is a constant, *λ*_1 _of a dominant trend test is a decreasing function of *p *while *λ*_0 _of a recessive trend test is an increasing function of *p*. This verifies the results in [12].

The fact that the VIF *λ*_0.5 _of the additive trend test doesn't depend on allele frequency of the candidate marker makes it possible that the *λ*_0.5 _can be consistently estimated from a sequence of unlinked markers with arbitrary allele frequencies [11]. Unfortunately this is not true for trend tests with *x *other than 0.5 since the quantity *λ*_*x *_does depend on allele frequency of the candidate marker. Therefore, dominant or recessive trend tests and other robust tests cannot be uniformly adjusted by the GC method using null markers with different allele frequencies. To overcome this problem, Zheng et al. [12] proposed to use null markers that have the same allele frequency as that of the candidate marker to evaluate the variance inflation factor. This constraint of matching allele frequency limits substantially the number of null markers that can be used.

### RGC method

In what follows, we propose a regression-based GC method to adjust for the frequency variability of null markers when the GC method is applied to the general trend tests and the Pearson chi-square test.

In the Appendix, we show that when cryptic relatedness is present, under the null hypothesis the variance of Δ_*x *_is a quartic polynomial of allele frequency *p*,(3)

Gorroochurn et al. [10] pointed out that when the population has several subpopulations, Δ_*x *_has a non-zero mean(4)

When the population is of pure CR, the theoretical value of *μ*_*x *_is zero. But in reality the PS and CR are usually mixed together, so it won't do any harm if we include this term in our analysis.

Let *B*_1_, *B*_2_, ..., *B*_*K *_be arbitrary *K *null markers with minor allele frequencies *p*_1_, ..., *p*_*K*_. For the *k*-th marker, let  be the genotype frequency estimate for genotype *j *in group *i*, *i *= 0, 1, *j *= 0, 1, 2. Then  is the analogue of Δ_*x *_for null marker *B*_*k*_. Let  be the sample estimate of *p*_*k*_. Then we estimate the coefficients in (4) by minimizing(5)

Denote the estimate of *α*_*i *_by , *i *= 0, 1, 2. Let . The estimates  of *β*_*i*_, *i *= 1, 2, 3, 4 in (3) can be calculated by minimizing(6)

Let *p *be the MAF of the candidate marker. Then we can estimate  and *μ*_*x *_by

The RGC-corrected Cochran-Armitage's trend test with score *x *can then be defined as(7)

The Cochran-Armitage's trend tests are more powerful than the Pearson's chi-square test if the genetic model or *x *can be correctly specified. When the genetic model is unknown and the score *x *may be subject to misspecification, robust tests such as Pearson's chi-square test is preferred. Zheng et al. [13] proposed the following 2-df Pearson's chi-square test(8)

where  is the estimate of the correlation coefficient of *Z*_0 _and *Z*_1_. Combining (7) and (8) together, we therefore, propose the RGC-corrected Pearson's chi-square test as(9)

### Simulation study

To assess the validity of the proposed RGC method, we have implemented extensive simulations. Following [11], we use Wright's coefficient *F *to measure the correlation due to CR. Since it is difficult to simulate pure CR data, following [11,12] and [14], we employ the following procedure to generate a CR population. Let *p *be the allele frequency of a marker. Assume that there are *L *subpopulations including *a*_1_,⋯, *a*_*L *_cases and *b*_1_, ⋯, *b*_*L *_controls. We first generate *p*_1_, ..., *p*_*L *_independently from the Beta distribution *Beta*((1 - *F*)*p/F*, (1 - *F*)(1 - *p*)/*F*). We then generate *L *subpopulations having allele frequency *p*_1_, ..., *p*_*L *_respectively, assuming that within each subpopulation Hardy-Weinberg equilibrium holds. Finally we mix the *L *subpopulations together. From long run, this mixed population would resemble a pure CR population.

The details of the data generation are as follows. We used two subpopulations in each of our simulation. First we chose an allele frequency *p *of a marker which could be either a candidate marker or a null marker. We generated each of *p*_1 _and *p*_2 _from the Beta distribution *Beta*((1 - *F*)*p/F*, (1 - *F*)(1 - *p*)/*F*). Let *C*_1_, *C*_2 _represent the two subpopulations. We calculated the probabilities *P*(*G*_*i*_|*C*_*j*_), *i *= 0, 1, 2, *j *= 1, 2 according to HWE. The disease prevalence *k*_*j *_in subpopulation *C*_*j *_was estimated by

We then calculated  and , the probabilities of genotype *G*_*i *_in cases and controls in subpopulation *C*_*j*_, by

Next we drew independent genotype counts (*a*_0*j*_, *a*_1*j*_, *a*_2*j*_) of cases and (*b*_0*j*_, *b*_1*j*_, *b*_2*j*_) of controls from multinomial distributions *Mul*() and *Mul*() respectively. We then mixed (*a*_0*j*_, *a*_1*j*_, *a*_2*j*_) and (*b*_0*j*_, *b*_1*j*_, *b*_2*j*_) up to obtain a case-control data set given in Table 1, with  and  for *i *= 0, 1, 2.

With this method of generating data, we simulated the cases of *p *= 0.2 and 0.45 where *p *is the minor allele frequency of candidate marker. The frequencies of unlinked null markers were selected randomly with equal probability from [0.1, 0.5]. The data for the *K *null markers with the same penetrances *f*_0 _= *f*_1 _= *f*_2 _and a candidate marker with different penetrances *f*_0_, *f*_1_, *f*_2 _are independently generated. The number of replicates in each simulation was 10, 000. To avoid the instability of the linear regression, the predictors were centered before to be fitted into the regression [15].

## Results

A regression-based genomic control (RGC) method is proposed and applied to association tests other than the additive trend test. This method allows for using arbitrary null markers in the GC correction procedure, in which the variability of the allele frequencies of the null markers is adjusted by linear regression. The method is assessed by extensive simulation results. In addition, the robustness of the proposed method to the errors in selecting null markers is evaluated. We also simulate the power of our method.

Table 2 provides simulated type I error results for the uncorrected, GC and RGC tests. It shows that the uncorrected trend tests have highly inflated type I error and the type I errors of GC-corrected test deviate from the nominal level 0.05 more or less. As can be seen from Table 2 the RGC tests yield almost all the simulated type I errors around 0.05. The only exceptions are when *p *= 0.2, *K *= 200 and *F *is either 0.01 or 0.02 the RGC-corrected *T*_0 _test yields p-values 0.063 and 0.065 respectively. This is because *T*_0 _uses the count of genotype *AA *only, therefore the sample size for this test is small.

**Table 2:** Type I error of the uncorrected and GC or RGC-corrected tests under *H*_0_: *f*_0 _= *f*_1 _= *f*_2 _(nominal level is 0.05, a = (500, 1500), b = (1500, 500).

*F*	MAF	*K*	Method	*T*_0_	*T*_1/2_	*T*_1_	
0.01	*p *= 0.2	200	Uncorrected	0.350	0.557	0.539	0.509
			GC	0.032	0.056	0.067	0.045
			RGC	0.063	0.054	0.052	0.055
		300	Uncorrected	0.335	0.551	0.532	0.497
			GC	0.027	0.047	0.057	0.038
			RGC	0.055	0.053	0.051	0.052
	*p *= 0.45	200	Uncorrected	0.446	0.543	0.486	0.496
			GC	0.085	0.051	0.035	0.053
			RGC	0.052	0.052	0.054	0.049
		300	Uncorrected	0.464	0.550	0.487	0.512
			GC	0.096	0.049	0.037	0.058
			RGC	0.051	0.050	0.052	0.051

0.02	*p *= 0.2	200	Uncorrected	0.473	0.667	0.650	0.633
			GC	0.026	0.046	0.061	0.040
			RGC	0.065	0.053	0.052	0.056
		300	Uncorrected	0.452	0.679	0.662	0.637
			GC	0.022	0.048	0.060	0.035
			RGC	0.054	0.050	0.051	0.053
	*p *= 0.45	200	Uncorrected	0.591	0.665	0.612	0.627
			GC	0.101	0.047	0.038	0.060
			RGC	0.052	0.053	0.054	0.050
		300	Uncorrected	0.581	0.663	0.610	0.622
			GC	0.106	0.047	0.032	0.062
			RGC	0.051	0.052	0.053	0.052

The frequencies of null markers are randomly selected from [0.1, 0.5].

Table 3 presents the simulated power of RGC-corrected tests. From this table, we see that the trend tests with the correct mode of inheritance have optimal power. The Pearson's chi-square test has less power but is very robust as to model specifications.

**Table 3:** Power of RGC-corrected tests- nominal level 0.05, *K *= 200, a = (300, 200), b = (200, 300).

*F*	MAF	Model	*T*_0_	*T*_1/2_	*T*_1_	
0.01	*p *= 0.2	DOM(*f*_0 _= 0.1, *f*_1 _= *f*_2 _= 0.15)	0.134	0.791	0.857	0.777
		ADD(*f*_0 _= 0.1, *f*_1 _= 0.17, *f*_2 _= 0.24)	0.401	0.805	0.781	0.734
		REC(*f*_0 _= *f*_1 _= 0.1, *f*_2 _= 0.2)	0.803	0.378	0.130	0.728
	
	*p *= 0.4	DOM(*f*_0 _= 0.1, *f*_1 _= *f*_2 _= 0.15)	0.179	0.704	0.852	0.780
		ADD(*f*_0 _= 0.1, *f*_1 _= 0.14, *f*_2 _= 0.18)	0.701	0.905	0.856	0.866
		REC(*f*_0 _= *f*_1 _= 0.1, *f*_2 _= 0.2)	0.995	0.936	0.418	0.990

0.02	*p *= 0.2	DOM(*f*_0 _= 0.1, *f*_1 _= *f*_2 _= 0.15)	0.129	0.682	0.767	0.674
		ADD(*f*_0 _= 0.1, *f*_1 _= 0.17, *f*_2 _= 0.24)	0.362	0.698	0.689	0.636
		REC(*f*_0 _= *f*_1 _= 0.1, *f*_2 _= 0.2)	0.753	0.333	0.130	0.687
	
	*p *= 0.4	DOM(*f*_0 _= 0.1, *f*_1 _= *f*_2 _= 0.15)	0.179	0.704	0.852	0.780
		ADD(*f*_0 _= 0.1, *f*_1 _= 0.14, *f*_2 _= 0.18)	0.655	0.853	0.811	0.809
		REC(*f*_0 _= *f*_1 _= 0.1, *f*_2 _= 0.2)	0.987	0.876	0.403	0.974

The frequencies of null markers are randomly selected from [0.1, 0.5].

Selection of null markers is an important issue when applying the GC method. The null markers are presumably unlinked to the disease, but in practice some linked loci may be chosen as null markers. To investigate the influence of the inclusion of linked markers in the set of null markers, we allowed the markers to be linked to the disease with probability 2%. Table 4 shows that the linked markers have some effect on the type I error which varies across genetic models. But the RGC method still controls the type I error around the nominal level 0.05.

**Table 4:** Type I error of the uncorrected, GC and RGC-corrected tests when the markers are linked to the disease with probability 2% (nominal level is 0.05, *K *= 200, a = (500, 1500), b = (1500, 500), *F *= 0.02, *f*_2_, *f*_1_, *f*_0 _are the penetrances for *AA, Aa, aa*.)

(*f*_0_, *f*_1_, *f*_2_)	MAF	Method	*T*_0_	*T*_1/2_	*T*_1_	
(0.01, 0.02, 0.02)	*p *= 0.2	Uncorrected	0.470	0.673	0.657	0.631
		GC	0.021	0.041	0.055	0.035
		RGC	0.064	0.051	0.047	0.058
(0.01, 0.015, 0.02)		Uncorrected	0.474	0.679	0.656	0.637
		GC	0.018	0.042	0.056	0.034
		RGC	0.056	0.052	0.051	0.054
(0.01, 0.01, 0.02)		Uncorrected	0.473	0.669	0.653	0.630
		GC	0.022	0.040	0.054	0.039
		RGC	0.063	0.052	0.053	0.055

(0.01, 0.02, 0.02)	*p *= 0.45	Uncorrected	0.592	0.668	0.615	0.630
		GC	0.098	0.046	0.034	0.062
		RGC	0.054	0.051	0.046	0.049
(0.01, 0.015, 0.02)		Uncorrected	0.608	0.675	0.619	0.638
		GC	0.105	0.045	0.033	0.060
		RGC	0.053	0.052	0.053	0.050
(0.01, 0.01, 0.02)		Uncorrected	0.598	0.670	0.620	0.631
		GC	0.103	0.045	0.034	0.061
		RGC	0.049	0.051	0.054	0.048

## Discussion

Case-control design is useful in detecting genes related to complex disease. For a case-control sample, if there is population structure and cryptic relatedness, spurious association between disease and genotype can occur due to variance inflation in the statistical tests. The genomic control method proposed by Devlin and Roeder [11] is a simple and effective method for eliminating spurious results caused by cryptic relatedness.

However when applying the GC method to correct for inflation of type I error of general trend test or the Pearson's chi-square test, it is required that the null markers are matched with the candidate marker in allele frequencies. This matching limits the applicability of the GC method. In this paper we propose a RGC method to correct for the population stratification effects which allows for use of any null markers. To adjust for the variability of allele frequencies of the null markers we estimate the inflated variance *τ*_*x *_and the noncentral parameter *μ*_*x *_by linear regression. This RGC method can be applied to the Cochran-Armitage's trend tests other than the additive trend test, with arbitrary score, the Pearson genotype-based association test and other robust efficiency tests.

Simulation results show that the RGC method can properly correct for the inflation of type I error of trend tests or Pearson's chi-square test caused by cryptic relatedness in the population. It is observed that the RGC method is slightly conservative for recessive trend test and anti-conservative for dominant trend test when the minor allele frequency is close to 0. We think that this is due to the instability of linear regression near the boundary of MAF values.

## Conclusion

Simulation studies show that the RGC method can effectively correct for the variance inflation caused by cryptic relatedness and is robust to inclusion of linked loci in the selection of null markers.

## Authors' contributions

TY carried out the implementation of the regression method, conducted all simulations and wrote the initial draft of the manuscript. YY developed the regression method and proposed the project. BH designed the study and wrote the final version of the manuscript. All authors read and approved the manuscript.

## Appendix

Here, we calculate the variance of Δ_*x *_under population structure and various genetic models. Assume that case-control samples come from *L *subpopulations, which include *a*_1_, ⋯, *a*_*L *_cases and *b*_1_, ⋯, *b*_*L *_controls, respectively. Thus ,  and *n*_0 _+ *n*_1 _= *n*. We also assume that individuals from different subpopulations are independent. For each subpopulation, the genotypic frequencies are described by(10)

where *p*_*i *_is the frequency of the allelic *A*_*i*_. Let

Using the results from Devlin and Roeder [11] and Zheng et, al [12], we have(11)(12)(13)

where *p *is the frequency of the allelic *A*.
